# Analysis of high dimensional data using pre-defined set and subset information, with applications to genomic data

**DOI:** 10.1186/1471-2105-13-177

**Published:** 2012-07-24

**Authors:** Wenge Guo, Mingan Yang, Chuanhua Xing, Shyamal D Peddada

**Affiliations:** 1 Department of Mathematical Sciences, New Jersey Institute of Technology, Newark, NJ 07102, USA; 2 Department of Mathematics, Central Michigan University, Mt. Pleasant, MI 48858, USA; 3 Department of Biostatistics, Boston University School of Public Health, Boston, MA 02118, USA; 4 Biostatistics Branch, National Institute of Environmental Health Sciences, Research Triangle Park, NC 27709, USA

## Abstract

**Background:**

Based on available biological information, genomic data can often be partitioned into pre-defined sets (e.g. pathways) and subsets within sets. Biologists are often interested in determining whether some pre-defined sets of variables (e.g. genes) are differentially expressed under varying experimental conditions. Several procedures are available in the literature for making such determinations, however, they do not take into account information regarding the subsets within each set. Secondly, variables (e.g. genes) belonging to a set or a subset are potentially correlated, yet such information is often ignored and univariate methods are used. This may result in loss of power and/or inflated false positive rate.

**Results:**

We introduce a multiple testing-based methodology which makes use of available information regarding biologically relevant subsets within each pre-defined set of variables while exploiting the underlying dependence structure among the variables. Using this methodology, a biologist may not only determine whether a set of variables are differentially expressed between two experimental conditions, but may also test whether specific subsets within a significant set are also significant.

**Conclusions:**

The proposed methodology; (a) is easy to implement, (b) does not require inverting potentially singular covariance matrices, and (c) controls the family wise error rate (FWER) at the desired nominal level, (d) is robust to the underlying distribution and covariance structures. Although for simplicity of exposition, the methodology is described for microarray gene expression data, it is also applicable to any high dimensional data, such as the mRNA seq data, CpG methylation data etc.

## Background

With the advent of high dimensional genomic data, researchers are able to study changes in the expression of several hundreds and thousands of variables such as genes or CpG’s under various experimental conditions (or phenotypes) in a given cell culture, tissue or an organism etc. Although identification of differentially expressed individual variables across experimental conditions is of general interest, in recent years there is considerable interest in analyzing sets of variables that belong to some pre-specified biological categories such as signaling pathways and biological functions. Numerous statistical and computational methods have therefore been developed for such analyses. Although the methods described in this paper are broadly applicable to any high dimensional data where the sets and subsets are pre-defined, for simplicity of exposition, we shall describe the methodology in the context of gene expression data. The available gene set analysis (GSA) methods can be broadly classified into two categories. Loosely speaking, the first category of methods, often referred to as competitive gene set methods, tries to answer questions such as “Given the collection of differentially expressed genes identified by a statistical/bioinformatics methodology, how enriched is a pre-specified set?” For example, suppose *S*_1_ and *S*_2_ are two pre-specified sets consisting of *N*_1_and *N*_2_genes respectively. Suppose an investigator identified a total of *n* genes, *n*_*i*_ of which belong to set *S*_*i*_*i* = 1, 2. Then this category of methods computes the probability of discovering *n*_1_(*n*_2_) or more genes from the set *S*_1_(*S*_2_). Several variations and innovations to Fisher’s exact test, Kolmogorov-Smirnov test, etc, have been proposed in the literature for obtaining the corresponding *p*-values (c.f. [1-5]). Software packages such as Ingenuity Pathway Analysis (IPA) report *p*-values using such tests. The second category of methods answers a different but equally important question (c.f. [4,6-11]), namely, “Is a given set of genes differentially expressed between two conditions?” In this category of methods the gene set information is directly used when selecting differentially expressed sets of genes between two experimental conditions and the question it answers has a clear biological meaning. Commonly this category of methods is referred to as self-contained methods, which is the focus of this paper.

Most earlier methods (belonging to either of the two categories described above) are based on univariate statistical tests and thus ignore the underlying dependence in the gene expression data (c.f. [1-5,9,12-14]). For a review one may refer to [11,15,16]. It is well known that univariate statistical methods for multivariate data may potentially increase false positive rate and/or decrease power [17].

A natural multivariate extension of the classical t-test is the Hotelling’s *T*^2^ test which can be used for comparing a set of genes between two experimental conditions. Consequently, several GSA methods using Hotelling’s *T*^2^ test have been proposed in the literature such as [18-20]. Intrinsically, the Hotelling’s *T*^2^ statistic requires the sample size to be larger than the number of variables. However, for GSA, it is common for the sample size to be much smaller than the number of genes in a set. As a consequence, the Hotelling’s *T*^2^ statistic is not uniquely defined. To deal with the singularity problem, several approaches have been proposed in the literature. For instance, Kong *et al*. [8] modified the Hotelling’s *T*^2^ statistic by replacing the inverse of sample covariance matrix by its Moore-Penrose inverse based on the first few eigenvalues. Although this procedure is appealing, there is arbitrariness in the choice of number of eigenvalues to be used. Recently [11] introduced a shrinkage based Hotelling’s *T*^2^ statistic by replacing the sample covariance matrix by a shrinkage estimator of the covariance matrix derived in [21]. Although such modifications are computationally more stable than the Hotelling’s *T*^2^, for large gene sets (i.e. sets with a large number of genes), they still pose computational challenges. It is because that the test statistic involves the inversion of a high dimensional covariance matrix even though it may be non-singular. Lastly, all multivariate methodologies described above implicitly assume that the gene expression data in the two experimental conditions are homoscedastic across all genes. That is, for a given set of genes the covariance matrix of gene expression in the two groups is identical. This, in our opinion, is a very restrictive assumption and may be hard to verify in practice when dealing with microarray data consisting of several thousands of genes.

To gain deeper understanding of the differences between the two experimental/test groups (e.g. cancer and normal patients), there is considerable interest in identifying not only sets of genes involved in a pathway or a biological process, but also in identifying subsets of genes belonging to a particular biological process within each significant set. For example, genes in the Vascular Endothelial Growth Factor (VEGF) pathway are important for angiogenesis. There are about 31 genes in this pathway that are involved in various biological processes. These 31 genes can be further partitioned into different subsets of biological functions and the biologist may be interested in discovering not only the VEGF pathway but also various subsets of genes within this pathway. For example, MAP2K3, MAP2K6, p38, MAPKAPK2, MAPKAPK3, and HSP27 are involved in Actin reorganization, FAK and Paxillin are involved in Focal Adhesion Turnover, whereas GRB2, SHC, SOS, Ras, Raf1, MEK1, MEK2, ERK1, and ERK2 are involved in gene expression and cell proliferation. Similarly, other genes in VEGF pathway are involved in various other biological processes, such as cell survival, vascular cell permeability, prostaglandin production, and nitric oxide production.

In examples such as the above, we may (i) be interested in using the additional information about the subsets to improve the power of detecting gene sets (such as the VEGF pathway), and (ii) not only be interested in knowing if genes in the VEGF pathway are differentially expressed between control and treatment group, but also interested in identifying subset of genes in biological processes within VEGF pathway that are also differentially expressed between the two groups. Methods described above and other multivariate statistical methods, such as the methods based on principal component analysis [7], the mixed effects logistic regression [6], analysis of covariance [10,22], are not designed to address such questions directly. If one ignores information regarding subsets, then there is not only a loss of biological information when interpreting the data, but also a potential loss in power. On the other hand, one may use the existing methods by taking the subsets as the unit of analysis rather than the sets. However, such a strategy destroys the underlying relationships among subsets within a set and consequently may result in loss of power.

In this paper we introduce a novel methodology that (a) is computationally simple and does not require inversion of any matrix, (b) exploits the underlying dependence structure, (c) is useful for identifying significant gene sets and subsets within each significant set, (d) controls the overall familywise error rate (FWER) at the desired nominal level, and (e) is robust to potential heteroscedasticity in the data.

The basic idea of the proposed method is rather simple. Using the available biological knowledge, we partition the sets of genes into various subsets within sets. Within each gene subset so obtained, we perform a variation of Hotelling’s *T*^*2*^test and calculate the corresponding *p*-value using bootstrap methodology. We then perform multiple testing corrections using Bonferroni method for controlling the FWER. To control the FDR, the proposed methodology can be easily modified by using Benjamini-Hochberg (BH) procedure [23] to replace the Bonferroni method. Using extensive simulations, we studied the performance of the proposed procedure in terms of power and the FWER control. We illustrate the proposed methodology using a recently published data of [24].

## Methods

### Notations

Suppose we are interested in comparing two experimental conditions on the basis of mean expression levels of genes belonging to *K* pre-specified sets of genes *S*_1_, *S*_2_,… *S*_*K*_. For instance, these gene sets may represent different pathways or biological functions, derived from databases such as GO, KEGG, IPA, etc. Furthermore, suppose each gene set *S*_*k*_, *k*=1,2,…,*K*, is a union of *m*_*k*_ pre-specified subsets *S*_*k*,1_,· · · ,*S*_*k*,*mk*_ such that $S_{k}=\overset{m_{k}}{\underset{i=1}{⋃}}S_{k,i}$. Note that $S_{k,i}⋂S_{k,j}$ is not necessarily an empty set for any *i*≠*j*. Suppose there are a total of *G* genes on the microarray and suppose **X**_*ij*_ is a *G*×1 random vector corresponding to the ^*jth*^sample, *j*=1,2,…,*n*_*i*_, in the ^*ith*^ group, *i*=1,2 with mean vector *E*(**X**_*ij*_)=***μ***_*i*_ and covariance matrix *Cov*(**X**_*ij*_)=***Σ***_*i*_, where ***μ***_*i*_=(*μ*_*i*1_,…,*μ*_*iG*_)^*″*^, *i*=1,2.

For set *S*_*k*_, we are interested in testing the following null and alternative hypotheses; *H*_*k*_:***μ***_1,*k*_=***μ***_2,*k*_ versus $H_{k}^{\prime \prime }$:***μ***_1,*k*_≠***μ***_2,*k*_, where ***μ***_*i*,*k*_=(*μ*_*i*,*j*_:*j*∈*S*_*k*_) denotes the mean vector of genes in the set *S*_*k*_for samples from the *i*th group, *i* = 1, 2. Similarly, for genes belonging to the subset $S_{k,j}\subset S_{k}$, the hypotheses of interest are, *H*_*k*,*j*_:***μ***_1,*kj*_=***μ***_2,*kj*_versus *H″*_*k*,*j*_:***μ***1,_*kj*_≠***μ***_2,*kj*_, where $\mu_{i,k_{j}}=(\mu_{i,l}:l\in S_{k,j})$ denotes the mean vector of genes in the subset *S*_*k*,*j*_ for samples from the *i*th group, *i*=1,2.

### The test statistic and its null distribution

We shall now describe the test statistic using a generic notation. Suppose, for *i* = 1, 2, **X**_*i*1_**X**_*i*2_,…, $X_{in_{i}}$ is a random sample of *G*×1 vectors from a common population with mean vector ***μ***_*i*_and covariance matrix ***Σ***_*i*_. Let ${\bar{X}}_{i}$ denote the sample mean vector corresponding to the ^*ith*^population, *i* = 1, 2, and let **S**denote the usual pooled sample covariance matrix. Samples randomly drawn from these two populations are independent. Then under the assumption of ***Σ***_1_=***Σ***_2_, the Hotelling’s *T*^2^ statistic is proportional to ${\left({\bar{X}}_{1}-{\bar{X}}_{2}\right)}^{\prime \prime }S^{-1}({\bar{X}}_{1}-{\bar{X}}_{2})$. For large values of *G*, statistics such as the Hotelling’s *T*^2^ and Fisher’s linear discriminant function can be unstable since they involve the inversion of a high dimensional covariance matrix **S**. In the context of discriminant analysis [25], it was surprisingly found that the linear discriminant function that ignored the off-diagonal elements of **S**performed better than Fisher’s linear discriminant function that used the entire matrix **S**. In addition, in practice it may not be suitable to assume that ***Σ***_1_=***Σ***_2_. Motivated by these reasons, we use the following test statistic for testing the null hypotheses described in the above subsection:

(1) $$\begin{array}{c} T_{\mathrm{diag}}^{2}\;=\;{\left({\bar{X}}_{1}\;-\;{\bar{X}}_{2}\right)}^{\prime \prime }{\left(\frac{\mathrm{Diag}(S_{1})}{n_{1}}\;+\;\frac{\mathrm{Diag}(S_{2})}{n_{2}}\right)}^{-1}\;\;\left({\bar{X}}_{1}-{\bar{X}}_{2}\right), \end{array}$$

where *Diag*(**S**_*i*_) is a diagonal matrix containing the diagonal elements of the sample covariance matrix **S**_*i*_*i* = 1, 2.

Since the underlying gene expression data are not necessarily multivariate normally distributed and the covariance matrices of these two groups are potentially unequal, the exact distribution of the above test statistic under the null hypothesis cannot be determined easily. We therefore adopt bootstrap methodology for simulating the null distribution of the test statistic such that the resulting methodology is not only robust to heteroscedasticity but also preserves the underlying dependence structure among genes. To do so, we draw simple random sample (with replacement) of *n*_*i*_ subjects from the *i*^*th*^group, *i* = 1, 2 and construct the bootstrap data using the residuals ${휖}_{\mathrm{ij}}^{*}=X_{\mathrm{ij}}-{\bar{X}}_{i}$*i* = 1, 2, *j*=1,2,…,*n*_*i*_ from the resampled subject *j*. Thus the bootstrap data are given by $X_{\mathrm{ij}}^{*}=\bar{\bar{X}}+{휖}_{\mathrm{ij}}^{*}$*i* = 1, 2, *j*=1,2,…,*n*_*i*_, where $\bar{\bar{X}}=\frac{n_{1}{\bar{X}}_{1}+n_{2}{\bar{X}}_{2}}{n_{1}+n_{2}}$, the weighted average of the two sample means, and ${휖}_{\mathrm{ij}}^{*}$ is the residual corresponding to the ^*jth*^subject selected. For more details regarding the residual bootstrap methodology we refer the reader to [26,27]. It is important to recognize that the residual bootstrap methodology implemented here is different from the usual bootstrap methodology. The standard bootstrap may not honor the structure present in the data and hence may potentially result in an inflated false positive rate. We remark that our proposed test statistic resembles the test statistic of [9], in the sense that neither procedure uses the off diagonal elements of the estimated covariance matrices. The two procedures, however, differ in the denominators used in the test statistic. The proposed test allows for unequal variances in the two populations that are being compared. Secondly, and more importantly, the two procedures fundamentally differ in the resampling schemes used. As noted above, the proposed methodology bootstraps the residuals and thus allows for any underlying dependence structure in the data (unknown to the user) whereas the resampling scheme used in [9] intrinsically assumes that under the null hypothesis the two populations under comparison are identically distributed, which is often not the case in practice. This is a major and an important difference between the two methods.

### The proposed strategy

For each $S_{k,j}\subset S_{k}$, let the test statistic (1) be denoted by $T_{\mathrm{diag},k,j}^{2}$ and let the corresponding bootstrap *p*-value be denoted by *P*_*k*,*j*_. If we have only a single gene-set *S*_*k*_with *m*_*k*_ gene-subsets *S*_*k*,1_,· · ·, $S_{k,m_{k}}$, then within *S*_*k*_, the problem of testing the significance of *S*_*k*,1_,· · ·, $S_{k,m_{k}}$is formulated as a problem of simultaneously testing a family of *m*_*k*_ null hypotheses, ${ℱ}_{k}=\{H_{k,1},\cdot \cdot \cdot \;,H_{k,m_{k}}\}$, using the available *p*-values *P*_*k*,*j*_’s. The gene-set *S*_*k*_ is declared to be significant if and only if at least one *H*_*k*,*j*_is rejected in the above problem of multiple testing.

There are two popular notions of type I error rates when dealing with the problem of simultaneously testing multiple hypotheses, one is to control FWER, which is the probability of falsely rejecting at least one true null hypothesis, and the other is to control the FDR, which is the expected ratio of false rejections to the total number of rejections [23]. In this article we shall only describe methods to control the FWER.

There are several FWER controlling procedures available in the literature for testing the family of null hypotheses, ${ℱ}_{k}=\{H_{k,1},\cdot \cdot \cdot \;,H_{k,m_{k}}\}$. In this paper we consider the following Bonferroni based procedure: For a given set of null hypotheses ${ℱ}_{k}$, we reject $H_{k,j}\in {ℱ}_{k}$ if *P*_*k*,*j*_≤*α*/*m*_*k*_. The corresponding Bonferroni-adjusted *p*-value for the set of null hypotheses ${ℱ}_{k}$ is $P_{k}^{*}=\min\{m_{k}P_{k,j},j=1,\ldots ,m_{k}\}$. Similarly, if we have multiple gene-sets *S*_*k*_,*k*=1…,*K*, each of which having *m*_*k*_gene-subsets *S*_*k*,1_,· · ·, $S_{k,m_{k}}$, then the problem of testing the significance of all gene-subsets in the *K* gene sets is formulated as a problem of simultaneously testing *K* families of null hypotheses, ${ℱ}_{k}=\{H_{k,1},\cdot \cdot \cdot \;,H_{k,m_{k}}\},k=1,\ldots ,K$, using the available *p*-values *P*_*k*,1_,…, $P_{k,m_{k}}$, *k*=1,…,*K*, in which for each gene-set *S*_*k*_,*k*=1,…,*K*, it is declared to be significant if and only if at least one hypothesis *H*_*k*,*j*_in ${ℱ}_{k}$ is rejected.

For testing the *K* families ${ℱ}_{k},k=1,\ldots ,K$, a simple Bonferroni based strategy is proposed as follows.

The Procedure

Step 1. Compute raw residual bootstrap *p*-value for each subset of genes.

Step 2. Compute adjusted *p*-values for each set *S*_*k*_(adjusting for the number of subsets within the set) as described above.

Step 3. Declare a set *S*_*k*_to be significant if its adjusted *p*-value is less than *α*/*K*. A subset *S*_*k*,*j*_within the set *S*_*k*_is declared to be significant if its raw *p*-value *P*_*k*,*j*_is less than *α*/*K*_*mk*_.

It is easy to see that the above proposed procedure strongly controls the overall FWER for any dependent test statistics, the probability of falsely rejecting at least one true null hypothesis in some family.

When the number of gene sets and gene subsets is large, it might be preferable to control the FDR rather than the FWER. The above proposed testing strategy controlling the FWER can be easily modified to control the FDR by using the BH procedure to replace the Bonferroni procedure when simultaneously testing the significance of the gene sets. Such modified strategy is very similar to a two-stage test strategy developed in [28] for controlling the overall FDR while selecting significant gene sets and their significant individual genes.

### Simulation study

We evaluate the performance of the proposed methodology in terms of power (the probability of rejecting at least one false null hypothesis) and the FWER control with Tsai and Chen’s method in [11], which uses the shrinkage estimator of the sample covariance matrix proposed in [21]. Note also that, unlike the bootstrap residuals used in the proposed methodology for deriving the null distribution of the test statistic, the resampling scheme used in [11] resembles the scheme used in [9]. Such resampling schemes do not honor the differences (if any) in dependence structure of the two populations that are being compared. Thus, if the two populations have different covariance structures under the null hypothesis, then as stated earlier in this paper, the standard permutation or standard bootstrap methodology can potentially result in an inflated FWER.

### Study design

In the simulation study, we considered two patterns of total number of sets of genes, which were 5 and 10. Since, in practice, the number of subsets and the number of genes within a subset may be unknown a priori, we allowed the number of subsets within each set of genes to be uniformly distributed in the range 5 to 16 and the number of genes within each subset was generated according to a uniform distribution in the range 5 to 10. To understand the robustness of the two methods in terms of FWER control, we considered a variety of probability distributions for the gene expression as follows: 

(1) Multivariate normal distribution, of appropriate dimension, with mean vectors **0**(for the control group) and ***μ***(for the treatment group), and covariance matrices ***Σ***_**1**_(for the control group) and ***Σ***_**2**_(for the treatment group), respectively. As commonly done, we assumed intra-class correlation structure for the two covariance matrices, with variances $\sigma_{1}^{2}$, $\sigma_{2}^{2}$ and correlation coefficients *ρ*_1_and *ρ*_2_, respectively. We considered two cases, namely, *σ*_1_=*σ*_2_, *ρ*_1_=*ρ*_2_(homoscedastic or homo.) and *σ*_1_≠*σ*_2_, *ρ*_1_≠*ρ*_2_(heteroscedastic or hetero.). In practice, one never knows a priori whether we have homoscedasticity or heteroscedasticity. Since genes within each subset (whether control or treated groups) may have different variances, for each gene we let *σ*_1_and *σ*_2_both take one of the five values, 0.1, 0.5, 1, 1.25 or 1.5, at random. Similarly, correlation coefficient between a pair of genes may not necessarily be constant across all subsets (whether control or treated groups), for each subset we let *ρ*_1_and *ρ*_2_both take one of the five values, 0, 0.25, 0.5, 0.75 or 0.9, at random. Thus the variance and correlation coefficients vary randomly from subset to subset. For each subset of genes, we always let mean vector ***μ***=**0**for the control group, ***μ***=*δ***1**for the treatment group, where *δ*was taken to be 0.5, 1 or 1.5 and **1**=(1,1,· · ·,1)^*″*^. For each group, we considered two patterns of sample sizes, namely, 10 and 40.

(2) Multivariate log normal distribution, where the vector of natural logarithm of each component follows multivariate normal distribution, with parameters as defined in the above setting of multivariate normal distribution, with *Σ*_1_=*Σ*_2_.

(3) Multivariate beta distribution. This distribution is motivated by CpG methylation data. Within each treatment group the multivariate beta vector was generated as follows. To generate *p* dimensional beta variable, we randomly generated *p* independent chi-square random variables *U*_1_,*U*_2_,…,*U*_*p*_with either 4 or 5 degrees of freedom and generated an additional independent chi-square random variable *V* with either 1 or 2 degrees of freedom. The resulting multivariate beta type random vector for a given treatment group is defined as **Z**=(*Z*_1_,*Z*_2_,…,*Z*_*p*_)^*″*^, where *Z*_*i*_=*U*_*i*_/(*U*_*i*_ + *V*), *i*=1,2,…,*p*. With the above choices of degrees of freedom, the mean methylation values (commonly called the “beta” value) for our simulated CpG’s ranged from approximately 0.67 to about 0.83.

(4) Mixtures of multivariate normal random vectors. For each treatment group we generated mixture of multivariate normally distributed data **Z**as follows:

(2) $$Z∼\mathrm{\Pi N}(0,\Sigma_{1})+(1-\Pi )N(1,\Sigma_{2}),$$

where *Π*=0.2. As in the case of multivariate normally distributed data in (1), we considered the homoscedastic as well as heteroscedastic covariance matrices for normal vector. The patterns of covariance matrices are as described in (1) above.

All our simulation results are based on a total on 1,000 simulation runs and 5,000 bootstrap samples.

## Results

In Table 1 we summarize the simulated FWER of the proposed Bonferroni method and the TS method. In all patterns considered the FWER of the proposed test was closer to the nominal level except in one case where the estimated FWER exceeded the nominal of 0.05 by one standard error. This corresponded to the mixtures of multivariate normal distributions case. On the other hand, as expected in the case of heteroscedastic data, the estimated FWER of the TS method often exceeded the nominal level of 0.05 by at least one standard error (which is approximately 0.007). Such cases are represented in bold face values. It is also interesting to note that the TS method was extremely conservative in the case when *n*=10 and the number of sets was 10. Although the shrinkage estimator of the covariance matrix is known to perform well for large *p* (the number of genes) and small *n* paradigm, in the present context as the number of sets of genes increases, the test statistic appears to be very conservative. This phenomenon is very striking when comparing the powers of the two tests (Table 2). The difference between the proposed method and TS is very noticeable especially when *δ* is close to the value assumed in the null hypothesis and when *n* =10, and the number of subsets is also 10. As we depart away from the null hypothesis, the TS method catches up with proposed test and there is very little difference between the two methods in terms of power for alternatives away from null hypothesis.

**Table 1:** **The simulated FWERs of the proposed method and the Tsai-Chen’s method at level*α*=0.05**


**Distribution**	**Variance**	**Sample**	**Number**	**Proposed**	**Tsai-Chen’s**
	**structure**	**size**	**of sets**	**method**	**method**
Normal	Homo.	*n*=10	5	0.027	0.019
			10	0.032	0.027
Normal	Homo.	*n*=40	5	0.047	0.054
			10	0.047	0.061
Normal	Hetero.	*n*=10	5	0.036	0.031
			10	0.044	0.021
Normal	Hetero.	*n*=40	5	0.038	0.066
			10	0.052	0.087
Log-Normal	Homo.	*n*=10	5	0.027	0.018
			10	0.024	0.022
Log-Normal	Homo.	*n*=40	5	0.048	0.039
			10	0.050	0.062
Mix. Normal	Homo.	n=10	5	0.018	0.009
			10	0.020	0.005
Mix. Normal	Homo.	n=40	5	0.055	0.050
			10	0.050	0.054
Mix. Normal	Hetero.	n=10	5	0.018	0.003
			10	0.017	0.003
Mix. Normal	Hetero.	n=40	5	0.058	0.060
			10	0.049	0.057
Multi. Beta	Var. func. mean	*n*=10	5	0.033	0.027
			10	0.031	0.031
Multi. Beta	Var. func. mean	*n*=40	5	0.043	0.042
			10	0.053	0.042

**Table 2:** **The simulated powers of the proposed method and the Tsai-Chen’s method at level*α*=0.05 for multivariate normally distributed data**

												
**Variance**	**Sample**	**Number**	***δ***	**Proposed**	**Tsai-Chen’s**												
**structure**	**size**	**of sets**		**method**	**method**												
Homo.	*n*=10	5	0.5	0.117	0.056												
		5	1	0.809	0.298												
		5	1.5	0.991	0.338												
Homo.	*n*=40	5	0.5	0.933	0.660												
		5	1	1.000	0.999												
		5	1.5	1.000	1.000												
Homo.	*n*=10	10	0.5	0.068	0.040												
		10	1	0.703	0.268												
		10	1.5	0.977	0.296												
Homo.	*n*=40	10	0.5	0.890	0.615												
		10	1	1.000	0.996												
		10	1.5	1.000	1.000												
Hetero.	*n*=10	5	0.5	0.147	0.037												
		5	1	0.842	0.188												
		5	1.5	0.997	0.222												
Hetero.	*n*=40	5	0.5	0.959	0.702												
		5	1	1.000	1.000												
		5	1.5	1.000	1.000												
Hetero.	*n*=10	10	0.5	0.090	0.029												
		10	1	0.743	0.164												
		10	1.5	0.988	0.181												
Hetero.	*n*=40	10	0.5	0.920	0.643												
		10	1	1.000	0.999												
		10	1.5	1.000	1.000												

We also compared the performance of the proposed procedure based on (1) with that based on the following Hotelling’s *T*^2^ type statistic which uses the entire sample covariance matrices **S**_1_ and **S**_2_

(3) $$T^{2}={\left({\bar{X}}_{1}-{\bar{X}}_{2}\right)}^{\prime \prime }{\left(\frac{S_{1}}{n_{1}}+\frac{S_{2}}{n_{2}}\right)}^{-1}\left({\bar{X}}_{1}-{\bar{X}}_{2}\right).$$

To ensure that the sample covariance matrices are non-singular, we chose the sample size in each group to exceed the total number of genes in each subset. In Table 3 we provide a small representative sample of simulation results. As can be seen from Table 3, the proposed procedure based on test statistic (1) has far greater power than the corresponding test based on (2) that uses the full sample covariance matrices. These findings, in the context of statistical testing, are consistent with [25] who discovered a similar phenomenon in the context of discriminant analysis for high dimensional data.

**Table 3:** Power comparison of the suggested testing strategy based on test statistic (1) and (2) for homoscedastic case and the number of genes = 20

	
**Number of**	***δ***	**Non-diagonal**	**Diagonal**	
**gene sets**				
5	0.5	0.298	0.637	
5	1	0.860	0.997	
5	1.5	0.998	1.000	
10	0.5	0.236	0.517	
10	1	0.780	0.993	
10	1.5	0.998	1.000	

### Illustration

Intramuscular injections among children often result in a variety of problems ranging from minor discomforts such as, rash and pain, to more serious complications resulting in emergency room visits [24]. Ferre *et al*. [24] conducted a gene expression study on a sample of 10 piglets to evaluate the effect of intramuscular injections on gene expression. Gene expressions were obtained at baseline, 6 hours, 2 days, 7 days and 21 days after injection. For details of the study design one may refer to [24]. To illustrate the proposed Bonferroni-based methodology we compared the mean expression of genes on day 7 with their mean expression at baseline. In all, there were 1,908 probes on the cDNA chip. Since the data on one of the pigs was missing for day 7, we only used data from 9 pigs in our paired analysis, where the expression (1) is suitably modified to reflect paired data. Using IPA we mapped these 1,908 probes onto 1,195 genes describing 75 biological categories. In Additional file 1: Table S1 (see online Supplementary Materials) we list all 75 biological categories along with their sub-categories. Note that the gene names and biological categories obtained from IPA are only meant for illustrating our methodology.

According to our Bonferroni-based methodology, 36 out of 75 biological categories are significant at FWER level of 0.05 (see Additional file 2: Table S2 in the online Supplementary materials). In Additional file 3: Table S3 in the online Supplementary Materials, we list sub-categories within each category along with their Bonferroni adjusted *p*-values. Results of the pathological examination of the injured muscle on day 21 (post injury) conducted by [24] revealed formation of dense fibrous and collagenous tissue in the area of injection with regeneration and maturation of myocytes throughout the injected area. A scar with new myofibers and connective tissue were formed. Relative to baseline, their individual gene expression analysis of day 21 revealed significant differential expression of genes such as collagens, fibronectin and matrix metalloproteinase, etc. Interestingly, such genes are involved in biological categories such as Genetic disorder, Skeletal and muscular disorder, Protein Synthesis, Cell morphology, Connective tissue development, and Cellular development, which were all significant sets according to our analysis (Additional file 2: Table S2). Our Bonferroni-based methodology allows a researcher to further probe the significance of each sub-category within the 36 significant categories. Results regarding the significance of each sub-category within each category are provided in the Additional file 3: Table S3 in the online Supplementary materials.

## Conclusions

Since biologists are often interested in identifying a collection of genes involved in a biological function or a pathway rather than individual genes, there has been considerable interest in recent years to develop statistical methods for identifying significant sets of genes. Usually, each pathway or biological function consists of a collection of (not necessarily disjoint) sub-pathways or sub-functions. Thus, each set of genes can be further partitioned into biologically meaningful subsets of genes. In this paper we exploit such structure information and propose a two-stage test strategy for selecting significant sets and subsets of genes between two experimental conditions while controlling the overall FWER. The proposed strategy is a general hierarchical test methodology, in which significant sets of genes are first identified by using Bonferroni procedure and then within each significant gene set, significant subsets of genes are further identified.

## Discussion

Although we do not discuss the problem of selecting significant gene sets and subsets when comparing multiple experimental conditions, the proposed methodology can be extended to such situations by replacing Hotelling’s *T*^2^ statistic by commonly used statistics such as the Hotelling-Lawley trace test or the Roy’s largest root test. Furthermore, if the experimental conditions are ordered, such as in a time-course or a dose-response study, one can exploit order-restricted inference based methods developed in [29]. As commented by a reviewer of this manuscript, it is possible that in some applications only a few genes in a given pathway are differentially expressed where such subsets are not necessarily pre-defined. We believe that discovery of such subsets could potentially generate interesting hypotheses for biologists to explore. The proposed methodology is targeted to identify pre-defined sets and subsets of genes that are differentially expressed, however, it would be interesting and useful to extend the proposed methodology to identify such unspecified subsets of genes.

## Competing interests

The authors declare that they have no competing interests.

## Authors’ contributions

WGG and SP conceived the study and developed the methodology. SP, MAY and CHX designed and performed the simulation studies. SP and CHX analyzed the data. SP, WGG, MAY and CHX wrote the manuscript. All authors read and approved the manuscript.

## Supplementary Material

Additional file 1 **Table S1.** Excel file containing gene sets, subsets and gene names. Click here for file

Additional file 2 **Table S2.** Excel file containing results of gene set analysis. Click here for file

Additional file 3 **Table S3.** Excel file containing results of gene subset analysis. Click here for file
